# Safflower petal composition: impact of sowing time and plant density on proximate, antioxidants, and colorants

**DOI:** 10.3389/fpls.2025.1711580

**Published:** 2025-11-21

**Authors:** Valeria Cafaro, Cristina Patanè, Vivienne Panebianco, Silvio Calcagno, Paolo Caruso, Giorgio Testa

**Affiliations:** 1 Institute of BioEconomy (IBE), National Research Council of Italy (CNR), Catania, Italy; 2 Department of Agriculture, Food and Environment (Di3A), University of Catania, Catania, Italy

**Keywords:** safflower petals, sowing time, plant density, antioxidants, carthamidin, carthamin

## Abstract

**Introduction:**

Safflower (*Carthamus tinctorius* L.) is a multipurpose plant that has recently attracted renewed interest as a natural source of antioxidant compounds and pigments from its petals, which can be used as alternatives to replace industrial compounds.

**Methods:**

In a split-plot experimental design, the effects of three winter sowings (December, January, and February) and two plant densities (D1, 25 plants m^−2^; and D2, 50 plants m^−2^) on petal production and composition in nutrients and antioxidants, including colorants, were examined in safflower (cv. Catima) in a semi-arid Mediterranean environment. Petals were harvested twice: at the flowering of the main shoots (early harvest) and 1 week later, and at the flowering of the lateral shoots (late harvest).

**Results:**

Petal production was reduced as sowing was shifted from December to February. The increase in plant density did not affect petals produced per plant but resulted in greater production per unit area. With sowing in January, crude protein was the highest (up to 17.2%), and oil content was the lowest (down to 3.72%). Total phenols significantly decreased (−6%) with the shift of sowing from December to February. Overall, they were accumulated to a greater extent at lower plant density (D1). Carthamidin (yellow pigment) was higher at the first harvest (up to 8.39%) and decreased thereafter, as carthamin (red pigment) was synthesized. Both pigments tended to decrease with the shift of sowing time and were lower in D1.

**Discussion:**

The nutritional value was positively associated with the nutraceutical value. Greater contents in phenols, flavonoids, and carthamidin, with minor changes in the proximate composition of petals, can be achieved with sowings in late fall–early winter. Harvest at late flowering (~90% flowers open on lateral shoots) resulted in greater yields and carthamin content but lower petal quality, suggesting that the choice of the harvest time of petals strictly depends on the specific trait desired.

## 1 Introduction

Safflower is an annual plant belonging to the Asteraceae family, native to the arid regions of the Middle East. Currently, although on a small scale, this plant is cultivated in more than 60 countries and regions worldwide, including Kazakhstan, the USA, Mexico, India, Turkey, and China, primarily for seed oil extraction, which is rich in bioactive compounds and highly polyunsaturated fatty acids (Buyukkurt et al., 2021). It is also used for food, industrial, and pharmaceutical applications (Vincent et al., 2024). In the past, safflower was cultivated for the extraction of colorants from its flowers for use in the textile industry (Kizil et al., 2008). Nowadays, the increasing demand for natural and sustainable colorants has renewed interest in safflower as a source of plant-based pigments. Safflower petals provide non-toxic water-soluble yellow pigment (carthamidin) and water-insoluble red-orange pigment (carthamin). The flowers of safflower are yellow-colored at early flowering, and they progressively change to red due to the conversion of yellow pigments to red pigment (Cho et al., 2000). Both pigments are currently used in food preparation to replace synthetic color additives or more expensive saffron (Steberl et al., 2020). Red pigment has also been proposed as a natural alternative to carcinogenic nitrate and nitrite in processed meat products (Kim et al., 2015). Furthermore, the growing demand for healthier and safer foods imposes a wider production of natural products rich in nutrients and antioxidants. In this regard, safflower petals have been proposed as natural plant sources of antioxidant compounds to replace industrial compounds (Ebadia et al., 2014).

In semi-arid Mediterranean regions, safflower can be cultivated either in spring or in winter. However, despite the great tolerance of safflower to drought conditions during growth (Yeilaghi et al., 2012), which makes this plant adapt to the dry farming systems of these areas, winter sowings may allow the crop to better exploit the soil water reserves formed during the rainy season and partially escape hot temperatures during flowering. These aspects make safflower a valid alternative to more commonly cultivated traditional crops. Nevertheless, safflower still receives limited attention and remains a minor crop.

Therefore, it is essential to promote the expansion and development of this still underutilized but economically important crop, with its petals being a natural source of eco-friendly compounds.

Agronomic management greatly affects the head and petal productions in safflower (Mohammadi and Tavakoli, 2015; Steberl et al., 2020; Al-Nafei and Al-Mohammad, 2021). Among the different agronomic factors, sowing time may largely influence the plant growth rate and, ultimately, final petal yield and composition (Attia et al., 2011; Patanè et al., 2020). Plant density also affects the rate of solar radiation interception and the level of plant competition for water and nutrient uptake, ultimately impacting the final yield and quality of the product (Al-Nafei and Al-Mohammad, 2021).

However, despite the importance of petals for colorants and bioactive compounds, most previous research has focused on seed and oil production. Very few studies have investigated how agronomic management, i.e., the combination of sowing time and plant density, affects petal yield, nutrient content, and bioactive compounds, including colorants. This represents a clear research gap that the present study aimed to address.

The objective of this field experiment, conducted in the framework of the EU H2020 project “MAGIC” (https://magic-h2020.eu), was to examine the combined effects of three winter sowings and two plant densities on head and petal productions and their composition in nutrients and bioactive compounds, including colorants, in a cultivar of safflower cultivated in a typical semi-arid Mediterranean environment.

## 2 Materials and methods

### 2.1 Experimental site and crop management

A field experiment was carried out in 2019 on the safflower cv. Catima (Semfor s.r.l., Verona, Italy) at the experimental station of the University of Catania (Italy), located on the Eastern coast of Sicily (South Italy, 10 m a.s.l., 37°25′N latitude, 15°30′ E longitude). The soil was a Vertic Xerochrepts soil and had the following characteristics: clay 28.3%, sand 49.3%, loam 22.4%, organic matter 1.4%, pH 8.6, total N 1.0‰, available P_2_O_5_ 5 mg g^−1^, and exchangeable K_2_O 245 mg g^−1^.

The experiment was arranged in a split-plot design with three replicates, where the effects of three sowing times (I, 17/12/2018; II, 21/01/2019; and III, 19/02/2019) and two plant densities [D1 (25 plants m^−2^) and D2 (50 plants m^−2^)] on plant head and petal production, and petals’ nutritional and antioxidant traits and colorants (carthamidin and carthamin), were assessed. Sowing time was assigned to the main plot and plant density to the sub-plot. The sub-plot had a size of 6 m^2^ (2 m long × 3 m wide). Plants were spaced 8 cm within rows, and 50 (in D1) or 25 cm (in D2) between rows. Before sowing, 100 kg ha^−1^ of P (as mineral superphosphate) and K (as potassium sulfate) were distributed. Nitrogen (100 kg ha^−1^ as ammonium sulfate) was distributed in a single application at sowing since fertilization as top dressing requires irrigation, and the plants of safflower in this study were grown under no irrigation following plant establishment.

One month after sowing, plants within rows were thinned to achieve the expected plant population. Approximately 400 m^3^ ha^−1^ of total water was distributed up to plant establishment. Afterward, irrigation was no longer applied to the crop. A manual weeding was performed once only, as the crop covered the soil, and the weeds could no longer grow.

### 2.2 Open field measurements

Throughout the crop-growing season, the main meteorological variables [daily maximum and minimum air temperature, rainfall, and evapotranspiration (ET)] were recorded using a data logger (CR10; Campbell Scientific, Inc., Logan, UT, USA) located ~50 m from the experimental field.

Heads were harvested twice per sowing date from all plants in the two internal rows. The first harvest was made on June 20 (plants of sowings I and II) and June 25 (plants of sowing III) 2019, when most of the plants reached the stage of flowering of the main shoot (codes 67–69 according to the BBCH scale; Flemmer et al., 2015). A second harvest occurred approximately 7–8 days later (on lateral shoots). At harvest, the number and fresh weight of the heads were measured. On a first subsample, the fresh and dry weight of the heads were measured, with the latter measured in a thermo-ventilated oven at 65°C until constant weight. On a second head subsample, petals were separated from heads, air-dried at room temperature (approximately 25°C) for 1 week in the dark (~8% moisture content), and weighed. The single head weight, number, and dry weight of total heads (per plant and per m^2^), and petal weight (per plant and per m^2^), were calculated.

### 2.3 Laboratory analyses

Samples of dry petals (approximately 30 g) from each plot were ground to fine powder in an electric mill and saved in a desiccator at room temperature (approximately 25°C) in darkness until use in laboratory analyses. Each sample was analyzed in triplicate.

#### 2.3.1 Crude protein

Crude protein content was measured in a BUCHI protein analysis system (BUCHI Italia, Assago, MI, Italy) using the Kjeldahl method (AOAC, 2005). The aliquots (0.5 g) of petal powder were transferred into a digestion tube containing 3 mL of 35% (w/w) H_2_O_2_, 6 mL of H_2_SO_4_/H_3_PO_4_, and a catalyst pill. After 1 h of digestion at 370°C and cooling to room temperature, the tube content was diluted with 30 mL of 33% (w/w) NaOH and 30 mL of distilled water; then, the tube was transferred to a distiller system. By distillation, ammonium hydroxide was trapped as ammonium borate into a 250-mL flask containing 160 mL of distilled water and 25 mL of a mixture made of 40% (w/v) boric acid solution, 0.1% (w/v) of bromocresol green solution in MeOH, and 7 mL of 0.1% (w/v) methyl red solution in MeOH, to a final 1-L volume with distilled water. Total N was determined by titration with 0.1 M HCl until the green color changed to pink. Crude protein content was calculated as follow (**Equation 1**):

(1) $$\text{Crude}\;\text{protein}\;\left(\%\right)=\frac{V\;\times \;M\;\times \;14.01}{W\;\times \;10}\;\times \;6.25$$

where *V* is the volume (mL) of HCl used for titration, *M* is the HCl molarity (0.1), 14.01 is the N atomic weight, *W* is the sample weight, 10 is the factor to convert mg g^−1^ to %, and 6.25 is the N to the protein conversion factor. Crude protein content was expressed on a dry weight (DW) basis.

#### 2.3.2 Oil

Oil content was determined according to the Randall method using a quantitative solvent extractor SER 148/6 (Velp Scientifica, Usmate Velate MB, Italy) (Randall, 1974). The aliquots (3 g) of the powdered samples were transferred into paper thimbles and extracted by immersion in *n*-hexane solvent at 130°C for 1 h. The extraction phase was followed by a washing phase for 1 h and a recovery phase for 25 min at 130°C. Glass vessels containing the extracted oil were placed in an oven at 100°C for 30 min to remove any solvent residues. After cooling, the vessels were weighed for oil content measurement. The oil content was calculated as follows (Cafaro et al., 2023) (**Equation 2**):

(2) $$\text{Oil}\;\text{content}\;\left(\%\right)=\;\frac{Wo}{W}\;\times \;100$$

where *W*o is the weight of the extracted oil and *W* is the sample weight. Oil content was expressed on a DW basis.

#### 2.3.3 Ash

The aliquots (2 g) of the powdered samples in porcelain crucibles were dried in an oven at 105°C and transferred to a furnace at 200°C, and then 300°C, 400°C, and 550°C at 1-h intervals, for a total of 3 h, before the samples became white. After that, crucibles were transferred to a desiccator for cooling. Ash content was calculated as follows (**Equation 3**):

(3) $$\;\text{Ash}\;\text{content}\;\left(\%\right)\;=\frac{W3-W1}{W2-W3}\;\times \;100$$

where *W*3 is the weight of the crucible and ash, *W*1 is the weight of the crucible, and *W*2 is the weight of the crucible with the sample. Ash content was expressed on a DW basis.

#### 2.3.4 Crude fiber

Crude fiber content was determined according to Van Soest et al. (1991) with modifications. The aliquots (1 g) of the powdered samples in glass crucibles were hydrolyzed in a hot extractor (Hanon analyzer mod. F800, Hanon Instruments, Jinan, Shandong Province, China) using a neutral detergent (ND). Samples were heated to boiling for 1 h, and then they were washed three times with boiling water and twice with acetone. Sample residues in crucibles were oven-dried at 105°C overnight, and then they were allowed to cool in a desiccator and weighed. After that, sample residues were ashed in a furnace at 550°C for ash content measurement and left to cool in a desiccator, and then they were weighed. Fiber content was calculated as follows (**Equation 4**):

(4) $$\text{Fiber}\;\text{content}\;\left(\%\right)\;=\frac{\;W1-W2}{W}\;\times \;100$$

where *W*1 is the weight of the crucible with sample residue at 105°C, *W*2 is the weight of the crucible with ash, and *W* is the sample weight. Crude fiber content was expressed on a DW basis.

#### 2.3.5 Total carbohydrates

Total carbohydrates were measured using the anthrone method (Önder et al., 2023) with modifications. The aliquots (5 mg) of the powdered samples were extracted in 4 mL of 1 M H_2_SO_4_, incubated in a hot water bath at 90°C for 1 h, and then centrifuged at 9,000 *g* for 10 min. After that, cold anthrone solution (4 mL) was added to 1 mL of extract, and all samples were incubated in a boiling water bath for 10 min; then, they were rapidly cooled in an ice bath. Absorbance was read spectrophotometrically at 630 nm using glucose (0 to 200 μg mL^−1^) as a standard curve (*R*^2^ = 0.99). Results were expressed as mg glucose g^−1^ DW.

#### 2.3.6 Total phenols

Total phenol (TP) content was measured using the Folin–Ciocalteu assay (Salem et al., 2011) with modifications. The aliquots (0.2 g) of the powdered samples were extracted in 10 mL of 80% MeOH. The extract was vortexed, incubated at room temperature overnight, and then centrifuged at 5,000 *g* for 20 min. After that, 200 μL of supernatant was mixed with 200 μL of the Folin–Ciocalteu reagent, and after a few minutes, 1.25 mL of a 7% (w/v) Na_2_CO_3_ solution and 1.5 mL of distilled water were added. All samples were incubated for 90 min at room temperature. Absorbance was read spectrophotometrically at 765 nm using gallic acid (0 to 250 μg mL^−1^) as the standard curve (*R*^2^ = 0.99). Results were expressed as mg gallic acid equivalent (GAE) g^−1^ DW.

#### 2.3.7 Flavonoids

Flavonoids were measured according to Ilahy et al. (2011) with modifications. The aliquots (0.2 g) of the powdered samples were extracted in 10 mL of 80% MeOH. The extract was vortexed, incubated at room temperature overnight, and then centrifuged at 5,000 *g* for 20 min. After that, 500 μL of the centrifuged methanolic extract was diluted in 1 mL of distilled water, and 90 μL of 5% (w/v) Na_2_CO_3_ was added. Samples were incubated for 6 min at room temperature, and 180 μL of 10% (w/v) AlCl_3_ was added. After 5-min incubation, 600 μL of 1 M NaOH and 630 μL of H_2_O were added to a final volume of 3 mL, and the mixture was vortexed. Absorbance was read spectrophotometrically at 510 nm using rutin (0 to 250 μg mL^−1^) as a standard curve (*R*^2^ = 0.99). Flavonoids were expressed as mg rutin equivalent (RE) g^−1^ DW.

#### 2.3.8 Carthamidin

Carthamidin was measured according to Mohammadi and Tavakoli (2015) with modifications. The aliquots (0.02 g) of the powdered samples were extracted in 100 mL of citric acid/disodium hydrogen phosphate buffer solution (pH = 5) overnight and then filtered. Carthamidin was determined spectrophotometrically at 400–408 nm. The percentage of carthamidin (*P*) was calculated as follows (**Equation 5**):

(5) $$P\;\left(\%\right)=\frac{A}{487}\times \;\frac{100}{W}\;$$

where *P* is the percentage of carthamidin, *A* is the maximum absorbance of the sample in the range of 400–408 nm, 487 is the specific absorbance of carthamidin, and *W* is the sample weight. Carthamidin content was expressed on a DW basis.

#### 2.3.9 Carthamin

Carthamin was measured according to Mohammadi and Tavakoli (2015) with modifications. The aliquots (0.02 g) of powdered petals were extracted in 100 mL of citric acid/disodium hydrogen phosphate buffer solution (pH = 5) overnight and then filtered (yellow pigment extraction). Residual petals were soaked in 100 mL of distilled water for 1 h, and this process was repeated three times. After that, residual petals were air-dried, then soaked in 15 mL dimethylformamide for 3 h, and filtered. Carthamin was determined spectrophotometrically at 525–535 nm. The percentage of carthamin (P) was calculated as follows (**Equation 6**):

(6) $$P\;\left(\%\right)=\frac{A}{992}\times \;\frac{100}{W}$$

where *P* is the percentage of carthamin, *A* is the maximum absorbance of the sample in the range of 525–535 nm, 992 is the specific absorbance of carthamin, and *W* is the sample weight. Carthamin content was expressed on a DW basis.

#### 2.3.10 Scavenging activity

The antioxidant activity of the safflower petal samples was measured using 2,2-diphenyl-1-picrylhydrazyl (DPPH) free radical scavenging assay, according to the method proposed by Hiramatsu et al. (2009) with modifications. The aliquots (0.2 g) of powdered petals were mixed with an 80% MeOH solution, vortexed, and incubated at 4°C for 30 min. After centrifugation at 3,000 *g* for 10 min, 1 mL of supernatant was mixed with 2 mL of freshly prepared 0.2 mM DPPH, vortexed, and incubated at room temperature in the dark for 30 min. Absorbance was read spectrophotometrically at 517 nm. A blank of 2 mL DPPH and 1 mL MeOH was used. Scavenging activity was calculated as follows (**Equation 7**):

(7) $$\text{Scavenging}\;\text{activity}\;\left(\%\right)=1-\frac{Abs517sample}{Abs517blank}\;\times \;100$$

### 2.4 Data analysis

Data on single head weight, total head and petal productions (per plant and unit area), and nutritional traits were statistically analyzed with a two-way analysis of variance (ANOVA) using the statistical software Minitab version 19, Minitab, LLC. ANOVA was carried out separately per harvest, considering *sowing time* (S), *plant density* (D), and their interactions as sources of variation. Before conducting the ANOVA, the normality of residuals was checked by means of the Shapiro–Wilk test and the homoscedasticity by means of Bartlett’s test. The independence of data was assumed by random sampling. Means were separated using Tukey’s test at a 95% confidence level.

Pearson’s linear correlation analysis (heatmap correlation) for the examined characters was performed using the statistical software Minitab version 19, Minitab, LLC.

A principal component analysis (PCA) was performed on petal production and all quality nutritional and nutraceutical traits using the statistical software Minitab version 19, Minitab, LLC. All data were distributed normally; therefore, they fulfilled statistical requirements for PCA.

## 3 Results

### 3.1 Weather conditions

**Figure 1:**
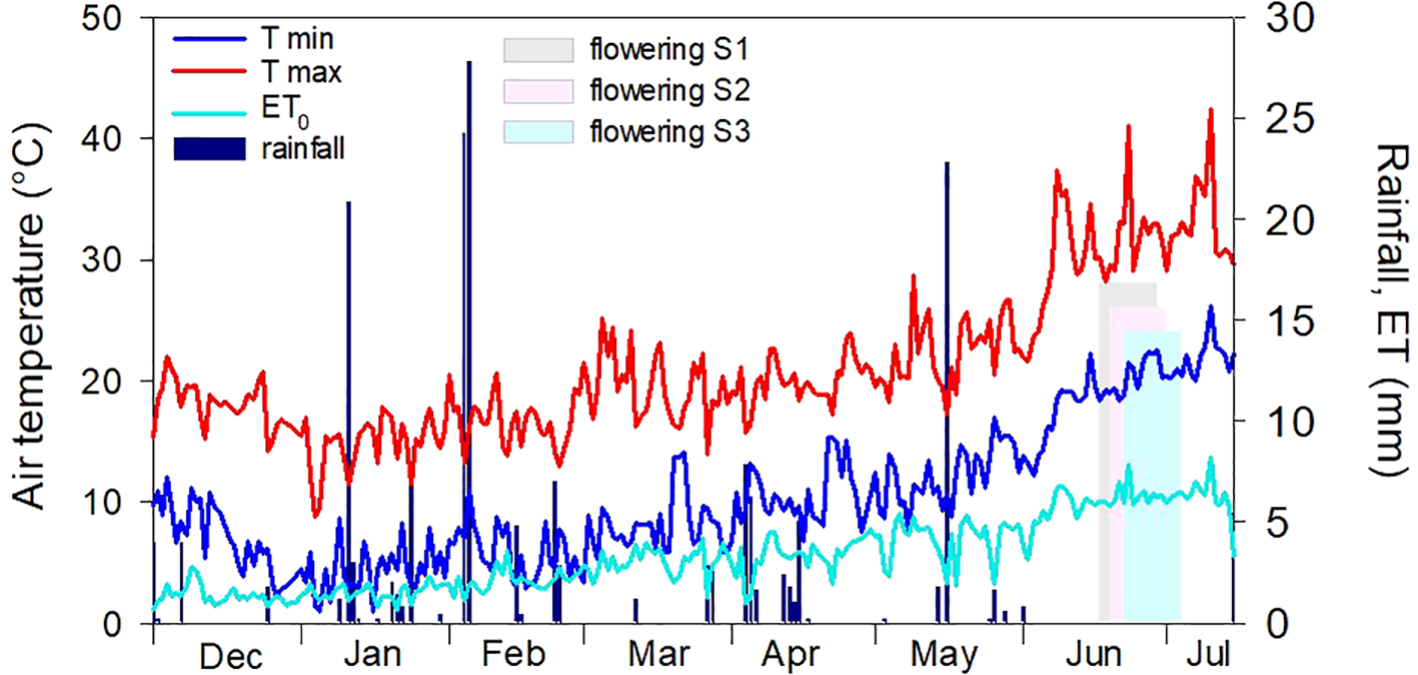
Meteorological course recorded during the field experiment (December 2018–July 2019). Light gray, pink, and blue bars indicate the extent of the flowering stage for the three sowing times (S1, 17/12/2018; S2, 21/01/2019; and S3, 19/02/2019).

The meteorological course recorded during the field experiment was that of a typical Mediterranean environment (**Figure 1**). Minimum temperatures ranged from 0.9°C to 22.5°C; maximum temperatures ranged from 8.8°C to 41°C. Mean temperature during flowering was approximately 1°C hotter, and the air was slightly drier (lower HR%, data not shown) for the plants of sowing III (that of February). Total rainfall during the experiment was 165.8 mm, with some rainy events in early February (for a total of 52 mm) and a single event (22 mm) in mid-May. Afterward, no relevant rainy events occurred until harvest. The average daily ET during the flowering period was 6.4 mm for all sowing times. Only a single event of high ET (7.85 mm) was recorded in late June, which corresponded to the highest value (41°C) of maximum temperatures recorded in the whole growth period.

### 3.2 Head and petal productions

The impact of sowing time and plant density on the production of heads and petals is detailed in **Tables 1** and **2**. The timing of sowing significantly influenced the number of heads per plant and per unit area. Notably, there was a marked decrease in the number of heads, in particular during the second harvest, which saw a reduction (up to −45%) per unit area when sowing was delayed from December to February (*S*, *p* < 0.01). The same trend was observed for total head weight, which was significantly reduced by 27% and 52% (first and second harvests, respectively) (weight per plant) and by 27% and 45% (first and second harvests, respectively) (weight per unit area), as sowing was delayed from early to mid-late winter.

**Table 1:** Effects of sowing time and plant density on head and floret productions at flowering in safflower cv. Catima (first harvest).

Experimental factors	Heads plant^−1^ (n)	Heads m^−2^ (n)	Heads plant^−1^ (g DW)	Heads m^−2^ (g DW)	Single head (g DW)	Petals plant^−1^ (g DW)	Petals m^−2^ (g DW)
Main effect							
Sowing time							
17/12/2018	9.48 ± 0.26a	354.2 ± 51.6a	13.50 ± 0.57a	501.3 ± 71.5a	1.42 ± 0.04 ab	1.00 ± 0.04a	37.18 ± 5.03a
21/01/2019	8.09 ± 0.42ab	299.1 ± 42.0ab	12.05 ± 0.72a	440.0 ± 54.7a	1.50 ± 0.07 a	0.80 ± 0.04b	29.04 ± 3.61b
19/02/2019	7.01 ± 0.55b	251.3 ± 28.5b	8.98 ± 0.85b	317.4 ± 31.9b	1.27 ± 0.04 b	0.75 ± 0.05b	26.89 ± 2.74b
Plant density							
25 plants/m^2^	8.66 ± 0.37	216.5 ± 9.3b	12.48 ± 0.69a	312.0 ± 17.3b	1.44 ± 0.05	0.92 ± 0.04a	22.94 ± 0.99b
50 plants/m^2^	7.73 ± 0.54	386.6 ± 27.0a	10.54 ± 0.91b	527.1 ± 45.5a	1.35 ± 0.05	0.78 ± 0.05b	39.14 ± 2.58a
Interaction effect							
Sowing I (17/12/2018)							
25 plants/m^2^	9.59 ± 0.56	239.9 ± 13.9cd	13.91 ± 0.96	347.8 ± 23.9b	1.45 ± 0.04	1.04 ± 0.08	26.12 ± 2.03cd
50 plants/m^2^	9.37 ± 0.13	468.5 ± 6.8a	13.10 ± 0.76	654.3 ± 37.9a	1.38 ± 0.06	0.96 ± 0.01	48.25 ± 0.42a
Sowing II (21/01/2019)							
25 plants/m^2^	8.43 ± 0.69	210.8 ± 17.3d	12.98 ± 1.19	324.6 ± 29.9b	1.55 ± 0.11	0.86 ± 0.01	21.43 ± 0.06d
50 plants/m^2^	7.75 ± 0.54	387.5 ± 27.0ab	11.11 ± 0.55	555.4 ± 27.6a	1.45 ± 0.11	0.73 ± 0.05	36.66 ± 2.72b
Sowing III (19/02/2019)							
25 plants/m^2^	7.95 ± 0.38	198.8 ± 9.6d	10.54 ± 0.62	263.6 ± 15.4b	1.33 ± 0.07	0.85 ± 0.01	21.26 ± 0.06d
50 plants/m^2^	6.07 ± 0.70	303.8 ± 34.9bc	7.42 ± 0.88	371.2 ± 44.2b	1.22 ± 0.01	0.65 ± 0.05	32.53 ± 2.41bc
Significance							
Sowing time (*S*)	**	**	**	***	*	***	***
Plant density (*D*)	ns	***	*	***	ns	**	***
*S* × *D*	ns	*	ns	*	ns	ns	*

For the interaction effects and the main effects, different letters, when present, within columns, indicate statistical differences at the 0.05 level (Tukey’s test). DW, dry weight. * ,**, and *** indicate significance at *p* ≤ 0.05, 0.01, and 0.001 levels, respectively; ns, not significant.

**Table 2:** Effects of sowing time and plant density on head and petal productions at flowering in safflower cv. Catima (second harvest).

Experimental factors	Heads plant^−1^ (n)	Heads m^−2^ (n)	Heads plant^−1^ (g DW)	Heads m^−2^ (g DW)	Single head (g DW)	Petals plant^−1^ (g DW)	Petals m^−2^ (g DW)
Main effect							
Sowing time							
17/12/2018	16.28 ± 0.65a	635.6 ± 98.3 a	11.18 ± 0.60a	445.6 ± 71.8a	0.68 ± 0.01	1.60 ± 0.04a	60.56 ± 9.54 a
21/01/2019	14.52 ± 0.43a	537.6 ± 73.1b	8.88 ± 0.79b	328.9 ± 49.5b	0.61 ± 0.04	1.20 ± 0.09b	45.97 ± 8.66b
19/02/2019	10.38 ± 1.47b	374.6 ± 52.4c	5.42 ± 0.29b	244.3 ± 39.0b	0.66 ± 0.04	0.68 ± 0.03c	29.66 ± 4.44c
Plant density							
25 plants/m^2^	14.43 ± 1.18	360.9 ± 29.6b	8.56 ± 1.07	238.1 ± 27.7b	0.64 ± 0.04	1.21 ± 0.14a	30.40 ± 3.42b
50 plants/m^2^	13.01 ± 1.08	671.0 ± 58.4a	8.42 ± 0.84	441.2 ± 46.5a	0.65 ± 0.02	1.11 ± 0.14b	60.39 ± 7.02a
Interaction effect							
Sowing I (17/12/2018)							
25 plants/m^2^	16.70 ± 0.42	417.4 ± 10.6bc	11.46 ± 0.39	286.5 ± 9.7bc	0.67 ± 0.01	1.64 ± 0.01	39.31 ± 1.96b
50 plants/m^2^	15.85 ± 1.32	853.8 ± 23.9a	10.90 ± 1.26	604.7 ± 19.9a	0.68 ± 0.02	1.57 ± 0.08	81.80 ± 0.32a
Sowing II (21/01/2019)							
25 plants/m^2^	15.05 ± 0.69	376.3 ± 17.4bc	9.20 ± 1.66	229.9 ± 41.5c	0.61 ± 0.09	1.28 ± 0.12	27.97 ± 3.58b
50 plants/m^2^	13.98 ± 0.41	699.0 ± 20.6a	8.56 ± 0.54	427.9 ± 26.9ab	0.61 ± 0.03	1.12 ± 0.14	63.96 ± 6.24a
Sowing III (19/02/2019)							
25 plants/m^2^	11.55 ± 3.04	288.9 ± 76.0c	5.02 ± 0.49	197.7 ± 73.3c	0.65 ± 0.07	0.71 ± 0.05	23.92 ± 7.76b
50 plants/m^2^	9.21 ± 0.50	460.3 ± 25.1b	5.82 ± 0.16	290.8 ± 8.1bc	0.66 ± 0.04	0.65 ± 0.03	35.40 ± 2.31b
Significance							
Sowing time (*S*)	**	***	***	**	ns	***	***
Plant density (*D*)	ns	***	ns	***	ns	ns	***
*S* × *D*	ns	*	ns	*	ns	ns	*

For the interaction effects and the main effects, different letters, when present, within columns, indicate statistical differences at the 0.05 level (Tukey’s test). DW, dry weight. * ,**, and *** indicate significance at p ≤ 0.05, 0.01, and 0.001 levels, respectively; ns, not significant.

Overall, plant density had a lower impact than the timing of sowing on both the number and weight of heads. While plant density significantly influenced the number of heads per unit area (*D*, *p* < 0.001), it did not affect the number of heads produced per plant (*D*, *p* > 0.05). In particular, more heads (up to +86% at the second harvest) per unit area were produced, as plant density was increased from 25 to 50 plants m^−2^. The same results were obtained in terms of total head weight per unit area, which was significantly higher (+69% and +85% at the first and second harvests, respectively) at 50 plants m^−2^. At the second harvest, a greater number of heads per plant and unit area did not compensate for smaller head size, and, as a result, head production was lower than that measured at the first harvest.

The two experimental factors significantly interacted on both the number and total weight of heads per unit area (*S* × *D*, *p* < 0.05). During both harvests, the number and weight of total heads per unit area were unaffected by the sowing time at the lowest plant density (25 plants m^−2^). However, at a higher density (50 plants m^−2^), these measurements were significantly lower for the last sowing (that in February).

The production of petals followed that of heads. As a result, total petal weight (both per plant and unit area) progressively decreased (*S*, *p* < 0.001) as sowing was delayed from December to February, according to the reduced number and weight of total heads. The losses in total petal weight (per unit area) with the shift of sowing time approached 51% (gap between sowings I and III) at the second harvest. For heads and petals, productions were maximized at high plant density (50 plants m^−2^) (*D*, *p* < 0.001).

A significant *S* × *D* interaction was found in total petal production per unit area. Indeed, while no changes with sowing time were observed at the lowest plant density, at 50 plants m^−2^, the production of petals was significantly maximized with the earliest sowing (that of December), at the first harvest, and with both sowings I and II (those of December and January, respectively), at the second harvest.

### 3.3 Nutrient content

#### 3.3.1 Crude protein

**Figure 2:**
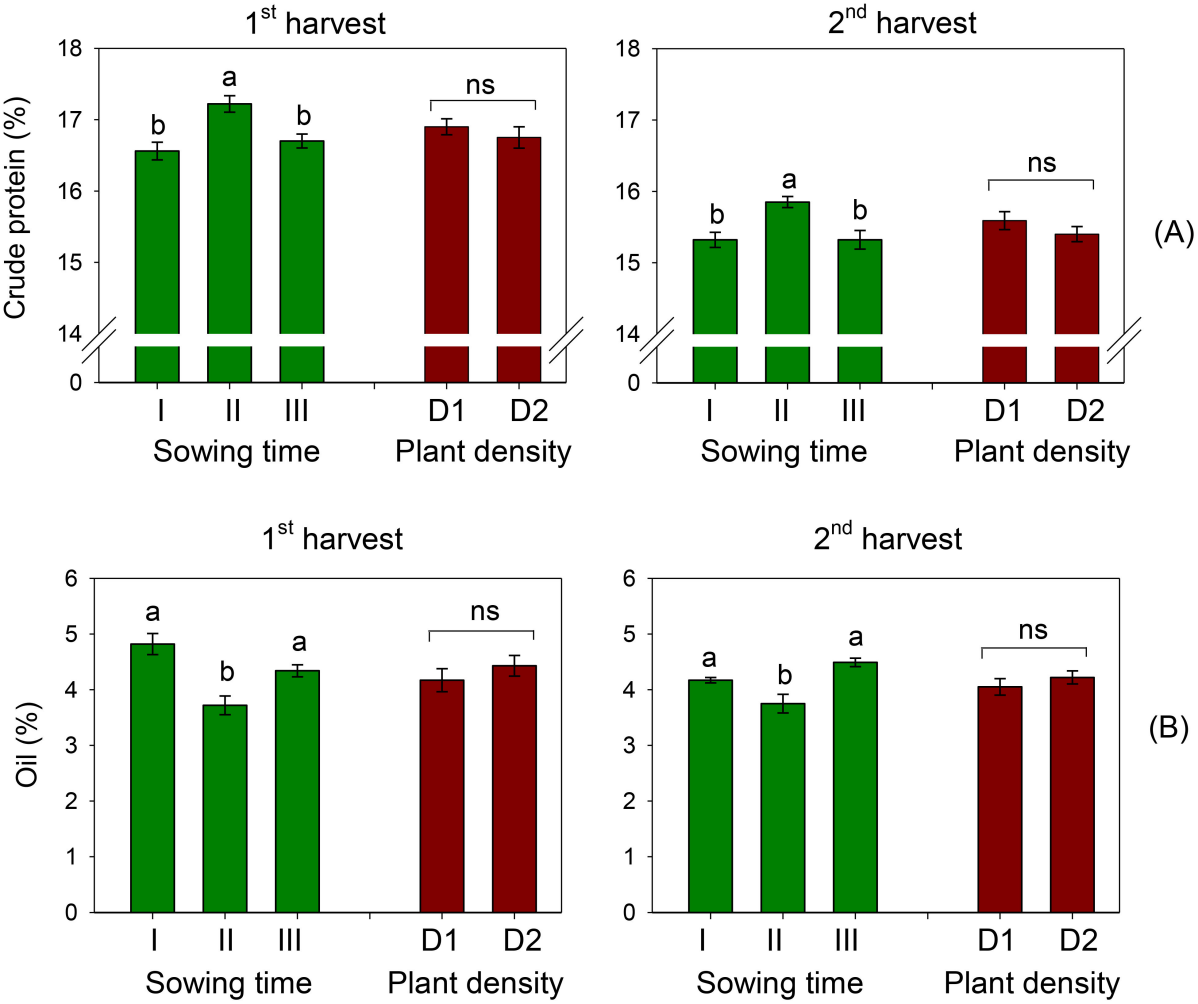
Mean effects of sowing time (I, 17/12/2018; II, 21/01/2019; and III, 19/02/2019) and plant density (D1, 25 plants m^−2^; and D2, 50 plants m^−2^) on crude protein **(A)** and oil content **(B)** in safflower petals (cv. Catima) at two harvest times. Different letters above bars indicate mean values statistically different at the 0.05 level (Tukey’s test). ns = not significant. Small black vertical bars indicate the standard error. Contents are expressed on a dry weight (DW) basis.

The crude protein content averaged 16.2% over the two harvests. This parameter was significantly influenced by the timing of sowing (*S*, *p* < 0.01), while plant spacing did not exhibit a significant effect (*D*, *p* > 0.05) (**Tables 3**, **4**). Across the two plant populations, the highest crude protein content (17.2% at the first harvest) was measured in petals of sowing II (that of January), and no differences were ascertained between contents in petals of sowings I and III (December and February, 16.6 and 16.7%, respectively) (**Figure 2A**). A one-week delay in harvest induced an average 8% decrease in crude proteins of petals. Sowing time and plant density did not interact on this trait.

**Table 3:** ANOVA results for nutritional traits in safflower petals in relation to sowing time and plant density (first harvest).

Source	Df	Crude protein	Oil	Ash		Crude fiber	Carbohydrates	
		*P*-value						
Sowing time (*S*)	2	0.005**	0.001**	0.004**	0.010**			0.117^ns^
Plant density (*D*)	1	0.309^ns^	0.183^ns^	0.028*	0.443^ns^			0.035*
*S* × *D*	2	0.923^ns^	0.701^ns^	0.455^ns^	0.689^ns^			0.343^ns^

Degree of freedom (df). * and ** indicate significance at *p* ≤ 0.05 and 0.01, respectively; ns, not significant.

**Table 4:** ANOVA results for nutritional traits in safflower petals in relation to sowing time and plant density (second harvest).

Source	df	Crude protein	Oil	Ash		Crude fiber	Carbohydrates		
		*P*-value							
Sowing time (*S*)	2	0.007**	0.002**		0.027*	0.002**	0.890^ns^		
Plant density (*D*)	1	0.152^ns^	0.103^ns^		0.676^ns^	0.029*	0.012*		
*S* × *D*	2	0.913^ns^	0.517^ns^		0.717^ns^	0.040*	0.251^ns^		

Degree of freedom (df). * and ** indicate significance at *p* ≤ 0.05 and 0.01, respectively; ns, not significant.

#### 3.3.2 Oil content

The oil content in petals of safflower was on average 4.29% and 4.14% at the first and second harvests, respectively. It was significantly affected by sowing time (*S*, *p* < 0.01) but not by row spacing (*D*, *p* > 0.05). A notable reduction in oil content was observed when sowing was changed from December to January, with a decrease of 23% at the first harvest and 10% at the second harvest (**Figure 2B**). Similar to the other traits examined, there was no interaction between sowing time and plant density on this trait.

#### 3.3.3 Ash content

**Figure 3:**
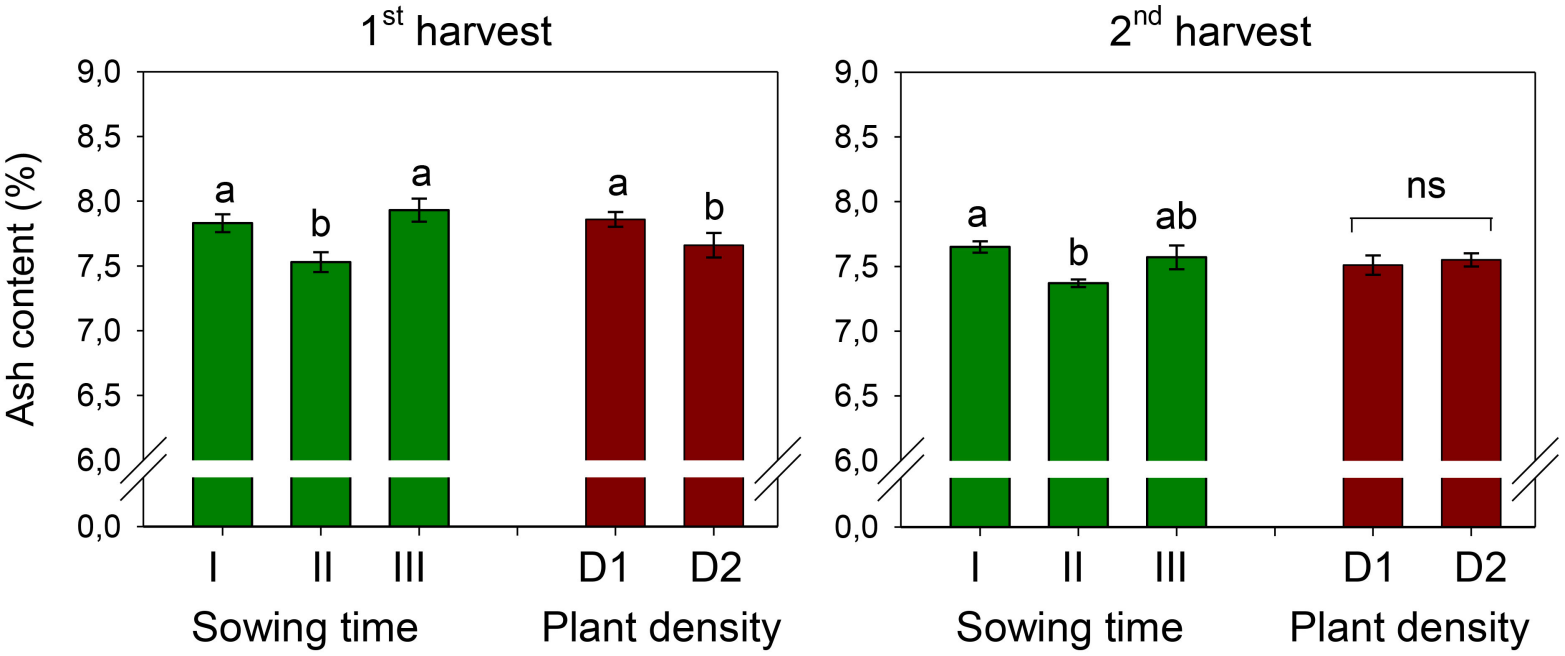
Mean effects of *sowing time* (I, 17/12/2018; II, 21/01/2019; and III, 19/02/2019) and *plant density* (D1, 25 plants m^−2^; and D2, 50 plants m^−2^) on ash content in safflower petals (cv. Catima) at two harvest times. Different letters above bars indicate mean values statistically different at the 0.05 level (Tukey’s test). ns = not significant. Small black vertical bars indicate the standard error. Contents are expressed on a dry weight (DW) basis.

Ash content measured at the first and second harvests was 7.76% and 7.53%, respectively (**Figure 3**). It was influenced by sowing time, being significantly lower (7.53% at the first harvest and 7.37% at the second harvest) in petals of sowing II (that of January). Plant spacing influenced the ash content of petals only at the first harvest (*D*, *p* < 0.05), where a 3% decrease occurred as the plant population increased from 25 to 50 plants m^−2^. The two experimental factors did not interact on ash content.

#### 3.3.4 Crude fiber content

**Figure 4:**
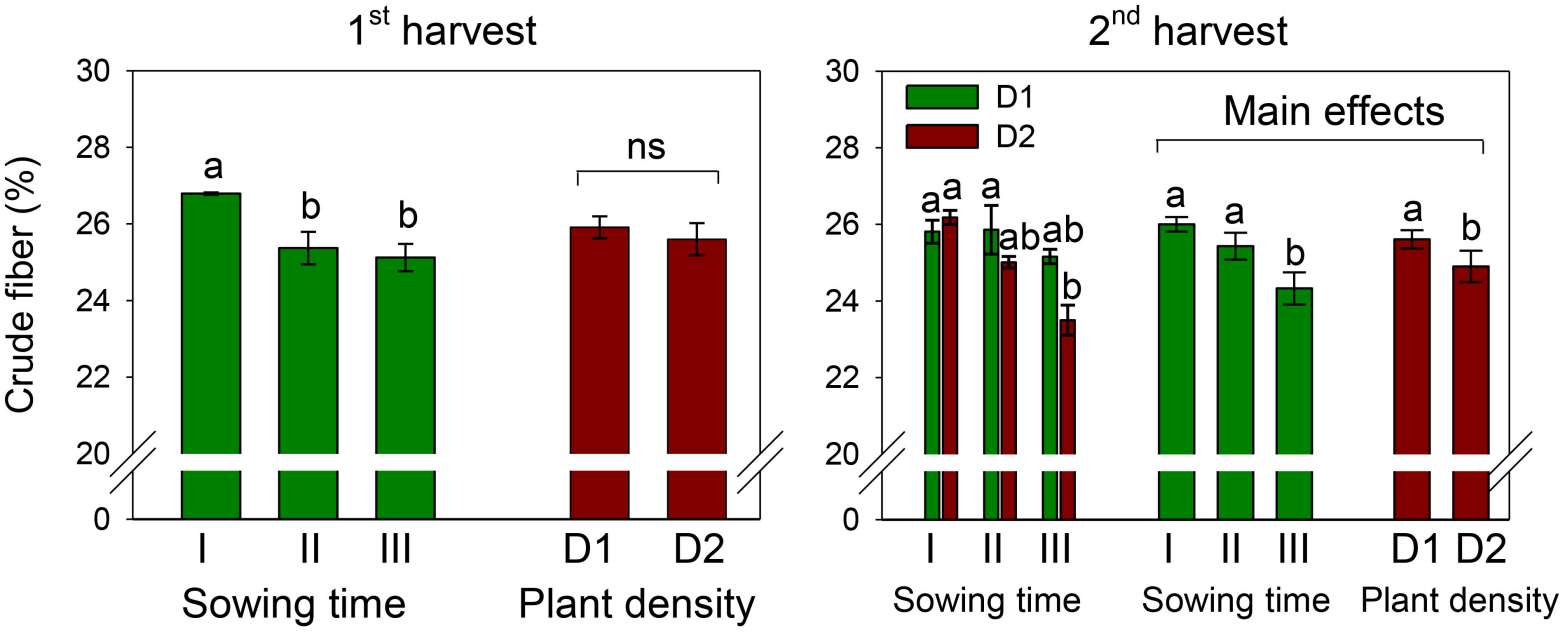
Mean effects of sowing time (I, 17/12/2018; II, 21/01/2019; and III, 19/02/2019) and *plant density* (D1, 25 plants m^−2^; and D2, 50 plants m^−2^) (first harvest) and interaction effect of *sowing time* (I, 17/12/2018; II, 21/01/2019; and III, 19/02/2019) × *plant density* (D1, 25 plants m^−2^; and D2, 50 plants m^−2^) (second harvest) on total crude fiber content in safflower petals (cv. Catima). Different letters above bars indicate mean values statistically different at the 0.05 level (Tukey’s test). ns = not significant. Small black vertical bars indicate the standard error. Contents are expressed on a dry weight (DW) basis.

The crude fiber content of petals was 25.8% and 25.3% at the first and second harvests, respectively, across sowing times and plant densities (**Figure 4**). Crude fiber was affected by sowing time, being the highest in petals of sowing I (that of December) and the lowest in petals of sowing III (that of February). At the first harvest, no effect of plant density and no interaction effect between the two experimental factors were ascertained on this trait.

Differently, at the second harvest, a significantly (*D*, *p* < 0.05) lower fiber content was measured in petals at high plant density (D2). Also, the two experimental factors slightly but significantly interacted on this trait (*S* × *D*, *p* < 0.05). While no difference among sowings was observed at low plant density (D1), at high density (D2), the fiber in petals of sowing I (that of December) was significantly higher (+10%) than that in petals of sowing III (that of February).

#### 3.3.5 Total carbohydrate content

**Figure 5:**
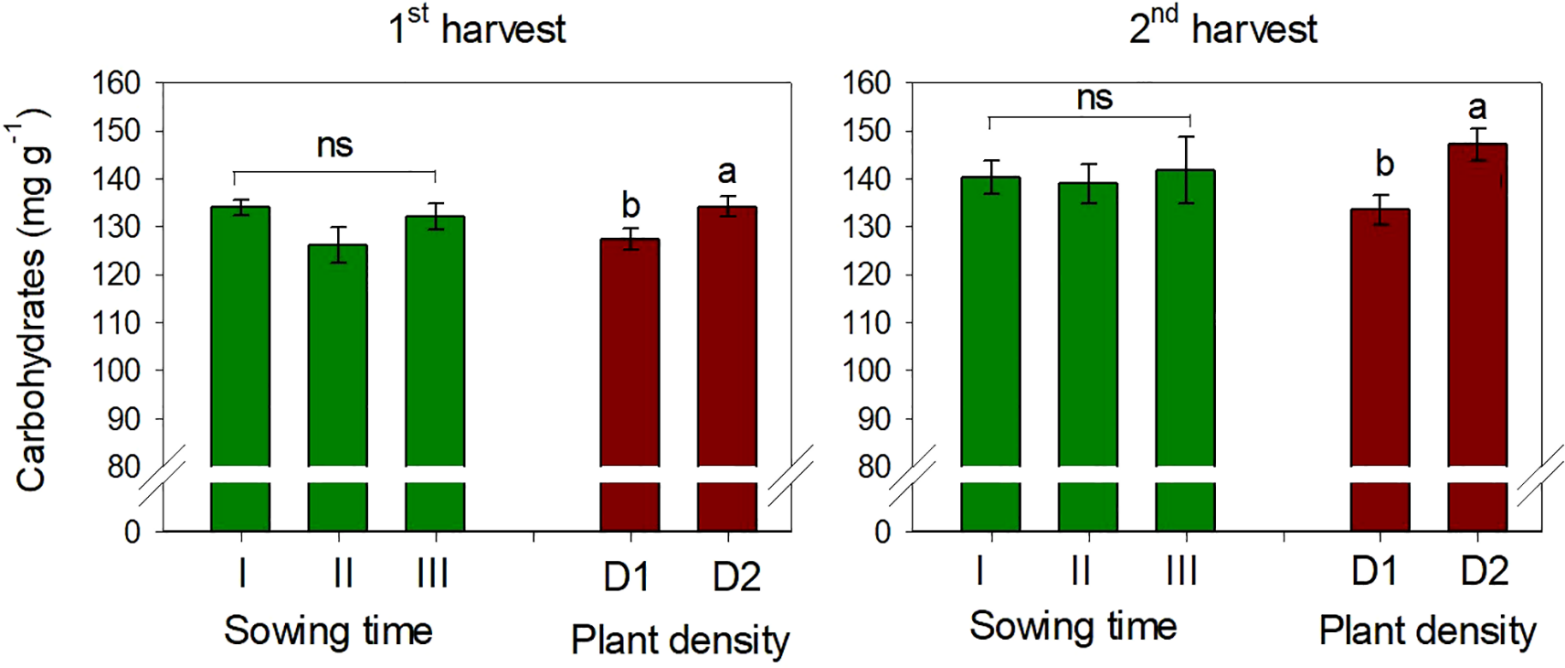
Mean effects of *sowing time* (I, 17/12/2018; II, 21/01/2019; and III, 19/02/2019) and *plant density* (D1, 25 plants m^−2^; and D2, 50 plants m^−2^) on total carbohydrate content in safflower petals (cv. Catima) at two harvest times. Different letters above bars indicate mean values statistically different at the 0.05 level (Tukey’s test). ns = not significant. Small black vertical bars indicate the standard error. Contents are expressed on a dry weight (DW) basis.

The content of total carbohydrates in safflower petals was on average 130.7 and 140.3 mg g^−1^ at the first and second harvests, respectively. It was slightly but significantly affected by plant density (*p* < 0.05), being +5% (first harvest) and +10% (second harvest) higher at low plant spacing (D2) (**Figure 5**). Sowing time had no effect on carbohydrate content, and no interaction *S* × *D* was observed on this trait.

#### 3.3.6 Total phenols and flavonoids

**Figure 6:**
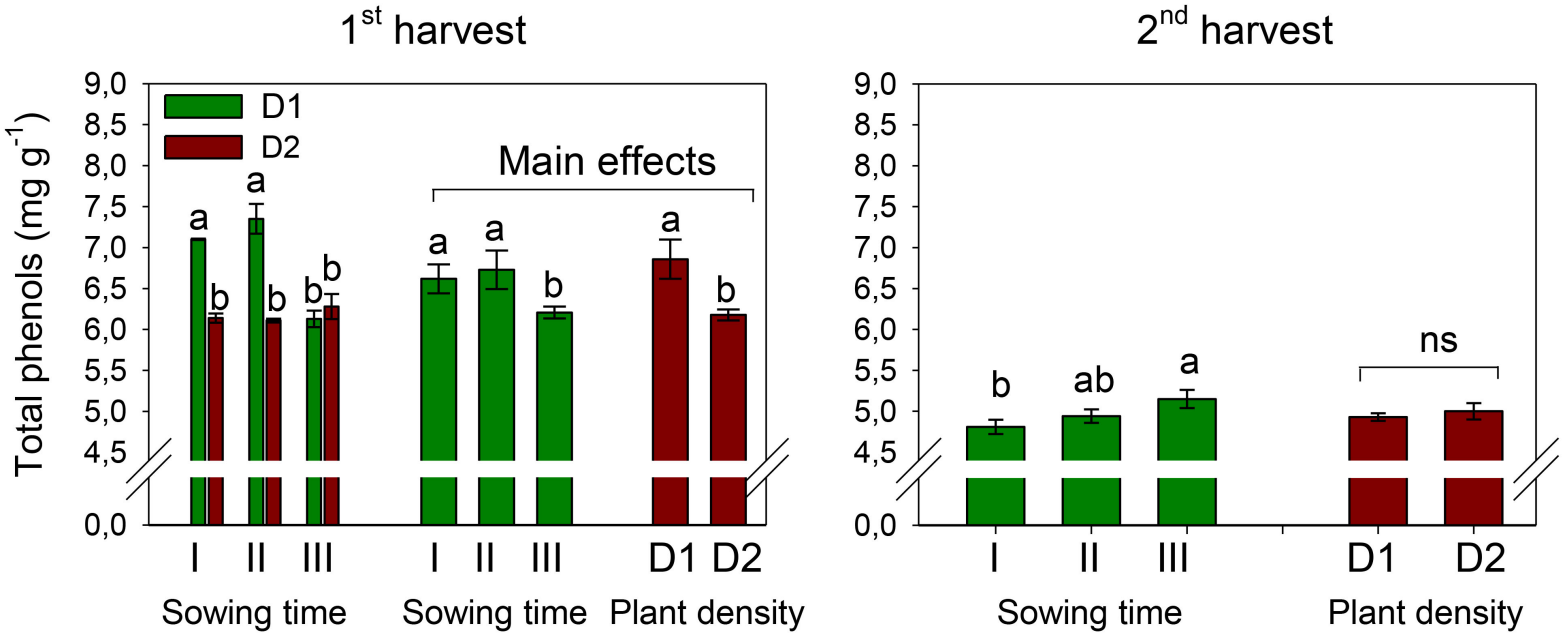
Interaction effect of *sowing time* (I, 17/12/2018; II, 21/01/2019; and III, 19/02/2019) × *plant density* (D1, 25 plants m^−2^; and D2, 50 plants m^−2^) (first harvest) and mean effects of *sowing time* (I, 17/12/2018; II, 21/01/2019; and III, 19/02/2019) and *plant density* (D1, 25 plants m^−2^; and D2, 50 plants m^−2^) (second harvest) on total phenol contents in safflower petals (cv. Catima). Different letters above bars indicate mean values statistically different at the 0.05 level (Tukey’s test). ns = not significant. Small black vertical bars indicate the standard error. Contents are expressed on a dry weight (DW) basis.

The total phenol contents of 6.52 and 4.97 mg GAE g^−1^, on average, were measured in petals at the first and second harvests, respectively (**Figure 6**). At the first harvest, TP slightly but significantly decreased (−6%) with the shift of winter sowing from December to February (*S*, *p* < 0.01) (**Tables 5**, **6**). Row spacing also influenced the level of TP in petals (*D*, *p* < 0.001), which was higher at greater plant density. The two experimental factors significantly (*p* < 0.001) interacted on TP content at the first harvest. Indeed, while with sowing in December and January, greater TP accumulated in petals at lower plant density, with sowing in February, TP achieved the same levels at both plant populations.

**Table 5:** ANOVA results for nutraceutical traits in safflower petals in relation to sowing time and plant density (first harvest).

Source	df	TP	Flav	Carthamidin	Carthamin	DPPH
		*P*-value							
Sowing time (*S*)	2	0.001**	0.023*	<0.001***	0.179^ns^	0.692^ns^
Plant density (*D*)	1	<0.001***	0.077^ns^	0.965^ns^	0.006**	0.009**
*S* × *D*	2	<0.001***	0.787^ns^	0.998^ns^	0.885^ns^	0.898^ns^

Degree of freedom (df).

TP, total phenols; Flav, flavonoids; DPPH, 2,2-diphenyl-1-picrylhydrazyl.

*, **, and *** indicate significance at *p* ≤ 0.05, 0.01, and 0.001, respectively; ns, not significant.

**Table 6:** ANOVA results for nutraceutical traits in safflower petals in relation to sowing time and plant density (second harvest).

Source	df	TP	Flav		Carthamidin	Carthamin		DPPH
		*P*-value							
Sowing time (*S*)	2	0.029*		0.064^ns^	<0.001***	0.027*	0.016*	
Plant density (*D*)	1	0.405^ns^		0.084^ns^	0.011*	0.029*		0.069^ns^	
*S* × *D*	2	0.327^ns^		0.425^ns^	0.682^ns^	0.691^ns^		0.601^ns^	

Degree of freedom (df).

TP, total phenols; Flav, flavonoids; DPPH, 2,2-diphenyl-1-picrylhydrazyl.

* and *** indicate significance at *p* ≤ 0.05 and 0.001, respectively; ns, not significant.

Different than what was observed at the first harvest, at the second harvest, the petals of sowing III were richer in TP than those of sowing I (*p* < 0.05). Row spacing did not significantly influence TP content during the second harvest, nor did the two experimental factors exhibit any interaction on this trait.

**Figure 7:**
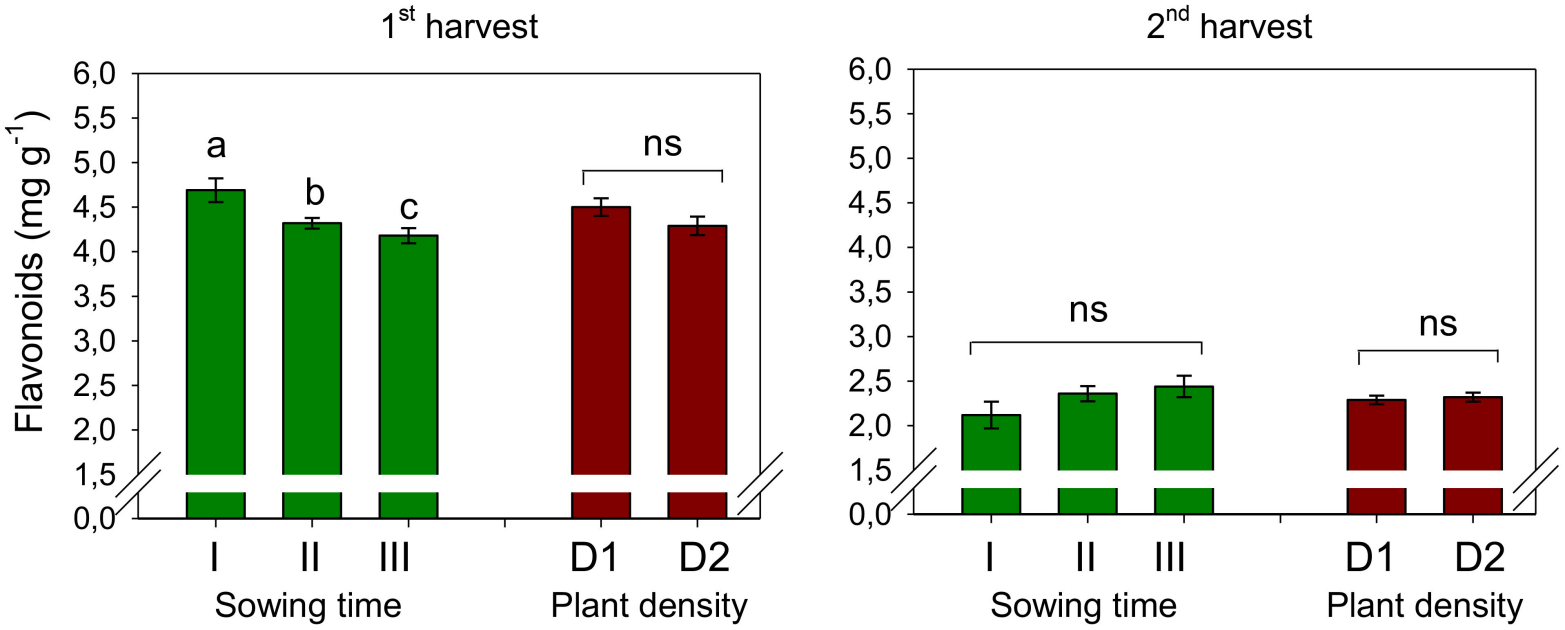
Mean effects of *sowing time* (I, 17/12/2018; II, 21/01/2019; and III, 19/02/2019) and *plant density* (D1, 25 plants m^−2^; and D2, 50 plants m^−2^) on flavonoid content in safflower petals (cv. Catima) at two harvest times. Different letters above bars indicate mean values statistically different at the 0.05 level (Tukey’s test). ns = not significant. Small black vertical bars indicate the standard error. Contents are expressed on a dry weight (DW) basis.

The average flavonoid content was measured at 4.40 mg RE g^−1^ during the first harvest and 2.31 mg RE g^−1^ during the second harvest, indicating a decline in flavonoid levels when petals were harvested 1 week later (**Figure 7**). Furthermore, at the first harvest, the flavonoid content experienced a significant decrease of 8% when sowing was delayed from December to January and a further decline of 11% when sowing was postponed to February (*S*, *p* < 0.01). Differently, at the second harvest, flavonoids did not change with sowing time. Plant population did not affect the flavonoid content, and no interaction was evidenced at ANOVA between the two experimental factors.

#### 3.3.7 Carthamidin and carthamin

**Figure 8:**
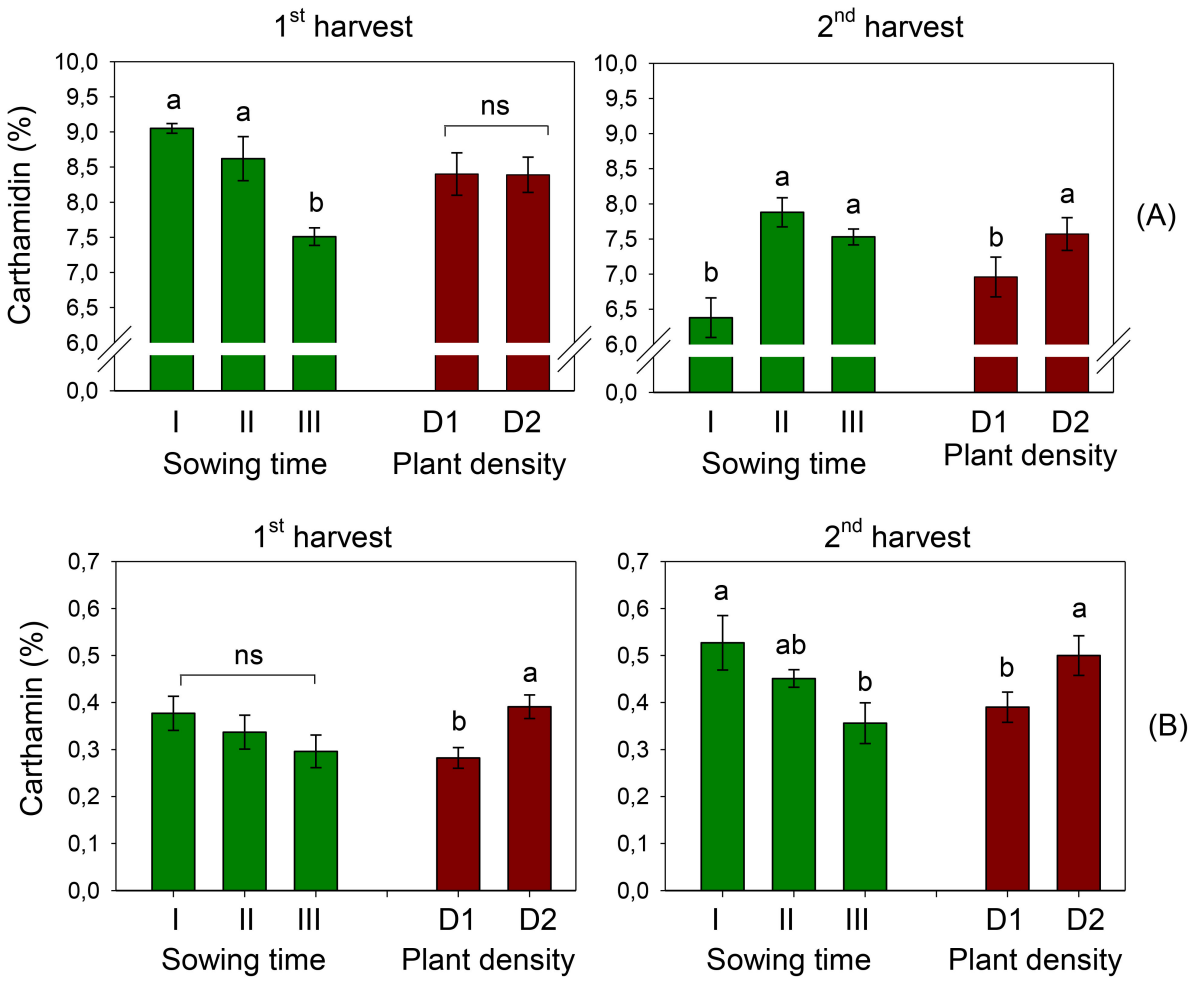
Mean effects of *sowing time* (I, 17/12/2018; II, 21/01/2019; and III, 19/02/2019) and *plant density* (D1, 25 plants m^−2^; and D2, 50 plants m^−2^) on carthamidin **(A)** and carthamin **(B)** contents in safflower petals (cv. Catima) at two harvest times. Different letters above bars indicate mean values statistically different at the 0.05 level (Tukey’s test). ns = not significant. Small black vertical bars indicate the standard error. Contents are expressed on a dry weight (DW) basis.

The content of carthamidin in safflower petals was 8.39% and 7.26% at the first and second harvests, respectively. It significantly varied with sowing time (*D*, *p* < 0.001) at both harvests (**Figure 8A**), although in a different way. At the first harvest, carthamidin exhibited a decreasing trend with the shift of sowing from December (sowing I) to February (sowing III, −17%), with no difference between the two plant populations. By contrast, at the second harvest, carthamidin in the plants of sowing I was significantly lower than that found in the plants of sowing III. Moreover, at the second harvest, a +9% carthamidin was measured in petals at high plant density. No effect of interaction *S* × *D* was evidenced at ANOVA on this trait.

Different than carthamidin, carthamin was higher when petals were harvested later, with averages of 0.34% and 0.45% at the first and second harvests, respectively. At the first harvest, carthamin content varied with plant density (*D*, *p* < 0.01), being significantly higher in D2; however, it did not change with sowing time. Differently, at the second harvest, carthamin content was also influenced by sowing time, and petals of sowing I (that of December) were richer in carthamin (0.53%) than those of the last sowing (that of February) (0.36%) (**Figure 8B**). For the first harvest, carthamin was greater at high plant density (+28%). No effect of interaction *S* × *D* was evidenced at ANOVA on this trait.

#### 3.3.8 Scavenging activity

**Figure 9:**
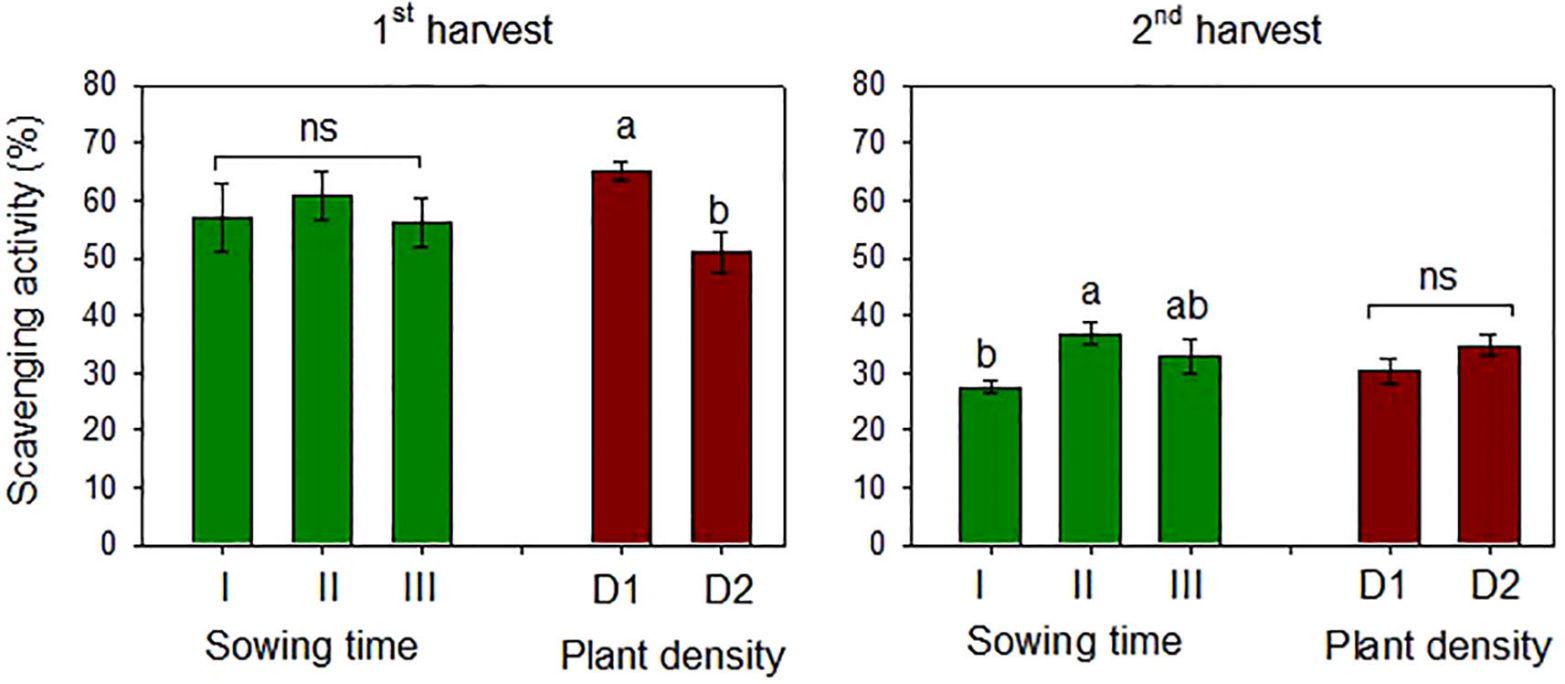
Mean effects of *sowing time* (I, 17/12/2018; II, 21/01/2019; and III, 19/02/2019) and *plant* density (D1, 25 plants m^−2^; and D2, 50 plants m^−2^) on scavenging DPPH radical activity (%) in safflower *petals* (cv. Catima) at two harvest times. Different letters above bars indicate mean values statistically different at the 0.05 level (Tukey’s test). ns = not significant; DPPH, 2,2-diphenyl-1-picrylhydrazyl.

The antioxidant activity of the safflower petals was estimated using the DPPH assay, which indicates the free radical scavenging capacity of the sample. Mean scavenging activities of 57.9% and 32.4% were measured at the first and second harvests, respectively. Safflower petals had higher antioxidant activity when sowing was postponed from December onward, although the differences were significant only at the second harvest (**Figure 9**).

Plant density influenced the antioxidant activity of the petals only at the first harvest, which was lower (−22%) at high plant density. No interaction between the two experimental factors was observed at ANOVA. The antioxidant capacity of the samples was higher in petals at the first harvest, according to the TP content measured at the same time.

### 3.4 Relationships

#### 3.4.1 Heatmap

**Figure 10:**
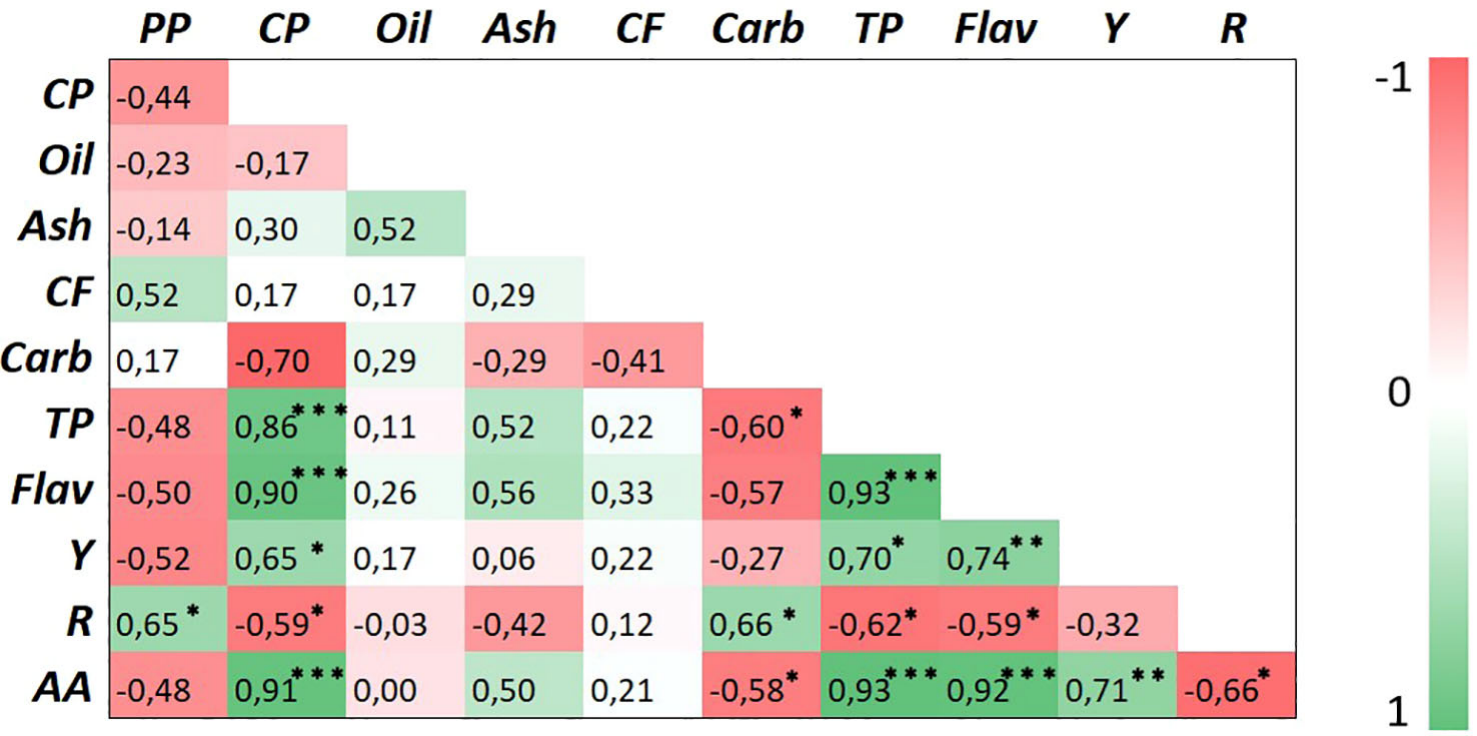
Heatmap of Pearson’s correlation matrix for the studied traits in safflower petals. Different abbreviations used in the figures are as follows: PP, petal production; CP, crude protein; CF, crude fiber; Carb, total carbohydrates; TP, total phenols; Flav, flavonoids; Y, yellow pigment (carthamidin); R, red pigment (carthamin); AA, antioxidant activity. *, **, and *** indicate significance at *p* ≤ 0.05, 0.01, and 0.001, respectively.

A heatmap correlation test was performed using all traits measured in safflower petals at both harvests (**Figure 10**). Petal production was positively correlated only with the red pigment carthamin (*p* < 0.05), but it was positively correlated with none of the other traits. Crude protein positively correlated with total phenols, flavonoids, and antioxidant activity (*p* < 0.001) and, less significantly, with carthamidin (*p* < 0.05), indicating that petals with high nutritional value in terms of protein content may also have high nutraceutical value. Contrastingly, crude protein content was negatively correlated with total carbohydrates and carthamin (*p* < 0.05). Interesting positive correlations were found for total phenols *vs*. flavonoids (*p* < 0.001) and carthamidin (*p* < 0.05). Among all nutraceuticals, the antioxidant activity positively correlated with total phenols and flavonoids (*p* < 0.001) and carthamidin (*p* < 0.01) and negatively with carthamin (*p* < 0.05). These results indicate that in safflower petals, the antioxidant activity was largely influenced by total phenols and flavonoids and, to a lesser extent, by the yellow pigment, but unexpectedly, was not associated with the carthamin content.

#### 3.4.2 PCA

**Figure 11:**
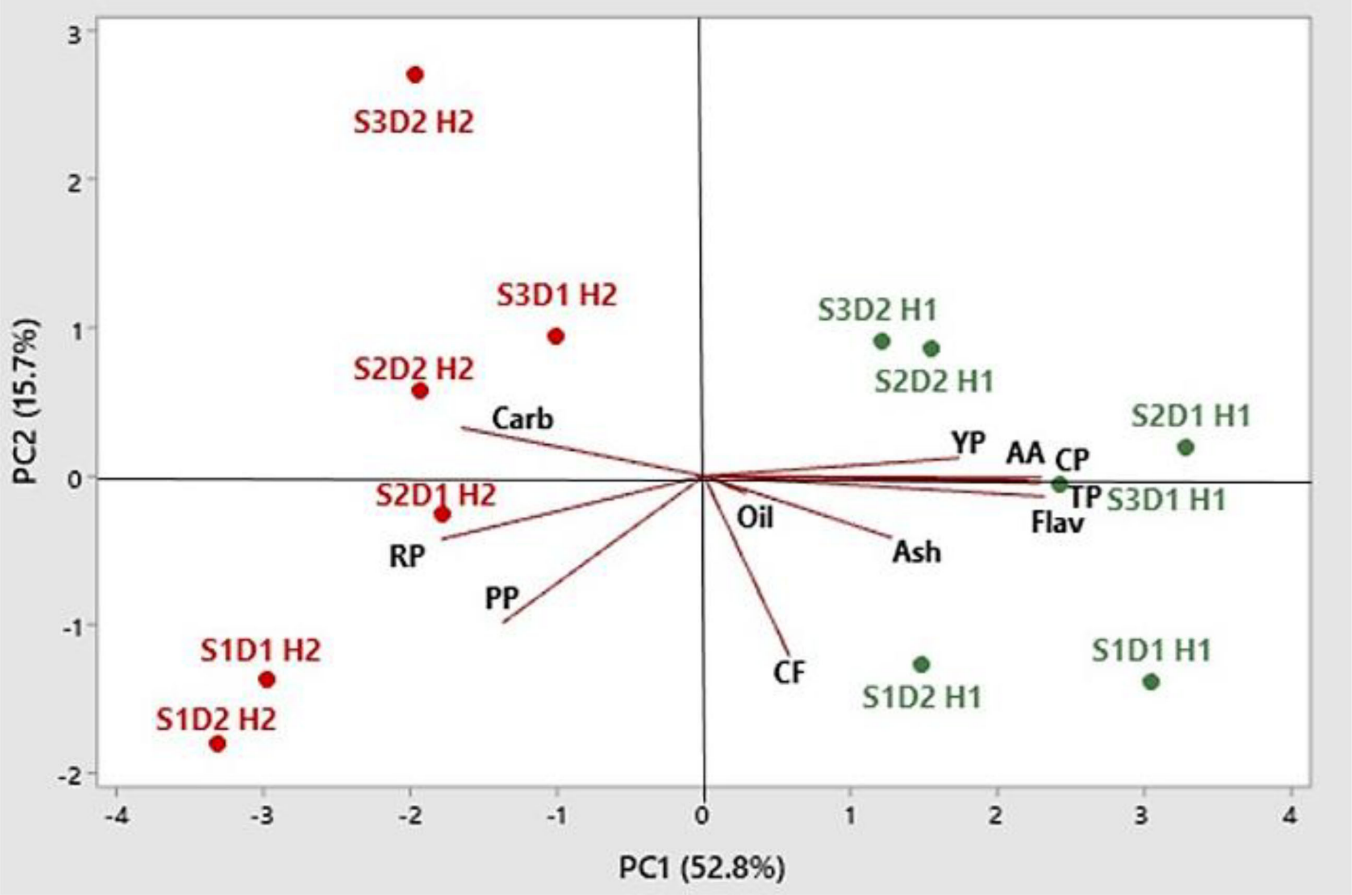
Principal component biplot and scores of PCA for petal production and nutritional and nutraceutical traits in safflower (S1, S2, and S3 indicate sowings I, II, and II, respectively; D1 and D2 indicate 25 and 50 plants m^−2^, respectively; H1 and H2 indicate first and second harvests, respectively; PP, petal production per plant; CP, crude protein; CF, crude fiber; Carb, total carbohydrates; TP, total phenols; Flav, flavonoids; YP, yellow pigment (carthamidin); RP, red pigment (carthamin); AA, antioxidant activity; PCA, principal component analysis).

A PCA was performed to evaluate the effects of the sowing time and plant density on the studied traits and to find interesting clusters (if any) within the experimental factors and the measured traits. PCA was carried out, including all data from both harvests. The analysis identified two factors (PC1 and PC2) that accounted for 68.5% of total variance, and they were used to score plots (**Figure 11**). Total carbohydrates, carthamin, and petal production per plant correlated positively, with the angle amplitude of their vectors <90°. Also, oil, ash, crude protein, total phenols, flavonoids, carthamidin, and antioxidant activity were correlated positively; however, changes in these traits were correlated negatively with changes in petal production, indicating that a high nutritional value of safflower petals is associated with a high nutraceutical value but also with low plant productivity in terms of petals.

The score plot analysis gave information on the changes in plant productivity and the nutritional and nutraceutical qualities of petals in safflower in relation to sowing time and plant density. PCA identified two distinct clustered main groups: the first group of sowings II and III (those in January and February) at both plant densities at the first harvest, in the upper quadrants on the right, which included crude protein, total phenols, carthamidin, and antioxidant activity, thus indicating high nutritional and nutraceutical values; the second group of sowing I (in December) at both plant densities and sowing II (in January) at low plant density, at the second harvest, including petal production per plant and carthamin content. Interestingly, all sowings and plant densities were positioned in the right part of the graph (positive value of PC1) with traits measured at the first harvest, while all sowings and plant densities were positioned in the left part of the graph (negative values of PC1) with traits measured at the second harvest.

## 4 Discussion

In this study, the effects of three winter sowing times and two plant densities on head and petal productions and nutritional traits were assessed in a cultivar of safflower cultivated in a typically semi-arid Mediterranean environment.

When winter sowing was shifted from December to February, head and petal plant productivity was significantly reduced as a probable effect of a shortening of the growth period (i.e., less biomass accumulated), although flowering onset was delayed (approximately 10 days later). The flowering of safflower is photoperiodically controlled (Torabi et al., 2020); therefore, plants in this study started flowering when photoperiod requirements were fulfilled, irrespective of sowing time. Photoperiod is an environmental factor that greatly affects the occurrence of flowering in many plants, and in long-day plants such as safflower, the flowering rate is accelerated as photoperiod increases (Torabi et al., 2020). The shift of sowing time also resulted in smaller heads. However, according to the findings of Emongor and Setshogela (2024), the number of heads per plant (*r* = 0.95** and 0.96**, harvest I and II, respectively), more than single head size (*r* = 0.47^ns^ and 0.22^ns^), influenced the petal production. Both head and petal productions were higher than those reported in literature for the same cultivar of safflower grown in the same environment with spring sowings in March and April, but matched those obtained in winter sowing in February (i.e., sowing III of the current study) (Patanè et al., 2020). These results indicate that winter sowings of safflower are more beneficial than spring sowings in terms of head and petal production.

However, other factors may influence head and petal productions in safflower. Among them, row spacing may have a major impact on light interception and plant growth, ultimately affecting plant production. Steberl et al. (2020) found that lower sowing density in safflower (40 plants m^−2^) resulted in a significantly greater number of branches and heads per plant as compared to higher density (75 plants m^−2^), leading to increased head and petal productions per plant. Differently, Al-Juheishy et al. (2024) observed that despite the wider row spacing (60 cm) determined an increased number of branches per plant, it did not result in higher heads per plant; these corroborate the findings of the present study, where the increase in plant density did not affect the number of heads produced per plant. Our findings also indicate that although the crop was no longer irrigated after plant establishment, plants at high density did not compete for the scarce available soil water during growth, confirming the drought tolerance largely recognized in safflower (Salem et al., 2014; Flemmer et al., 2015). However, more plants per unit area (50 plants m^−2^) resulted in greater production at a high plant population.

Petal production at the second harvest was higher than that measured at the first harvest. According to Steberl et al. (2020), this could be ascribed to the flowering of secondary and tertiary branches, which contributed to total plant production. Petal productions of 18.3–31.1 g m^−2^ were reported by Kizil et al. (2008) in different cultivars of safflower cultivated in Turkey during the winter–spring period, which are comparable to those obtained at the first harvest and much lower than those measured at the second harvest in this study.

In addition to head and petal productions, some quality traits of petals were assessed in safflower in response to sowing time and plant density. Indeed, literature on the effects of crop management on proximate composition and nutritional aspects of petals is still lacking.

In this study, a greater crude protein content (approximately 17% and 16% at the first and second harvests, respectively) corresponded to sowing II (that of January). Petals at the second harvest had fewer proteins than those at the first harvest at all sowing times and plant densities. A progressive loss of proteins during bloom was also reported in petals of *Chrysanthemum coronarium* L. as a result of a change in equilibrium between the rate of synthesis and degradation of particular proteins (Elanchezhian and Srivastava, 2001).

Oil content in petals changed with sowing date following an inverse trend of that of crude protein content, as also revealed by the significant negative relationship (*r* = −0.90* and −0.94** for the first and second harvests, respectively). Plant density did not affect this trait. Oil content measured in this study was within the range reported by Srinivas et al. (1999) and Erbaş and Mutlucan (2023) in safflower petals and higher than the contents reported by Emongor and Setshogela (2024) in both winter and summer sowings. Moreover, different than what was reported by Emongor and Setshogela (2024), who found a progressive increase in the oil content of petals during flowering from the onset to post-pollination, oil content did not change with harvest time. High α- and γ-linoleic acid contents indicate the beneficial effect of this oil on human health (Erbaş and Mutlucan, 2023). Important antifungal and antiviral activities have also been recognized in safflower petals based on the fatty acid composition of their oil (Srinivas et al., 1999).

Sowing in February resulted in fewer phenols and flavonoids in petals at the first harvest. Thus, sowings in early-mid winter (December and January in this study) may be beneficial for safflower petals in terms of nutraceutical content. Total phenols measured in this study were much higher than the contents reported by Buyukkurt et al. (2021) in safflower cultivated in Turkey. The role of bioactive compounds such as phenolics as a defense mechanism to counteract the damage of abiotic stresses, including thermal and water stress, has been largely proven (Siracusa et al., 2012; Salem et al., 2014). Indeed, in the present study, safflower plants experienced no rainfall and high temperatures during flowering, which may have accounted for high phenols and flavonoids in petals. Safflower is reported as a drought-tolerant plant able to modulate its phenolic content to face the oxidative stress caused by water limitation (Salem et al., 2014). Higher phenol contents in safflower petals were reported in literature at high plant density (Al-Nafei and Al-Mohammad, 2021), as a probable response of the plant to a competition stress for light, water, and nutrients. In this study, plant population did not affect the content of both phenols and flavonoids. However, different than what was reported by Al-Nafei and Al-Mohammad (2021), who changed within-row distances to manage plant density, in this experiment, we changed between-row distances while keeping the within-row distances. Djeridane et al. (2006) stated that the great phenol content typical of Asteraceae is related to the difficult climatic conditions (high temperatures, solar exposure, salinity, and drought) of their habitat, which stimulate the biosynthesis of bioactive compounds such as polyphenols. According to Salem et al. (2011), who reported a peak of phenols in safflower petals at the first stage of flowering and a sharp decrease afterward, in this study, higher phenol contents were measured at the first harvest, suggesting that early harvest (flowering of main shoots) is beneficial to maximize the content of these secondary metabolites. Antioxidants have been widely adopted as an additive to protect food against oxidative damage (Salem et al., 2011).

In this study, 8.4% and 7.3% of carthamidin content were measured in petals at the first and second harvests, respectively. This content satisfactorily matched that reported by Steberl et al. (2020) in safflower cultivated in Germany during springtime and was slightly higher than the content (approximately 7%) found in safflower grown in winter in Botswana (Emongor and Setshogela, 2024). According to Salem et al. (2014), dry conditions such as those that occurred in the current study during flowering may have resulted in an increase in secondary phytochemicals, including colorants. Lower carthamidin in the plants of the last sowing (that in February) indicated that a delayed sowing depressed the biosynthesis of the yellow pigment carthamidin in safflower petals at the first harvest. However, at the second harvest, the carthamidin measured in petals of sowing I (that of December) was lower than that of petals of the following sowings (II and III). According to Emongor and Setshogela (2024), carthamidin content is maximized at the start of flowering and decreases thereafter because carthamin is synthesized from the yellow precursor (Mohammadi and Tavakoli, 2015). In the current study, the plants of sowing I were probably at a slightly advanced stage of flowering with respect to those of sowings II and III, which may be responsible for lower carthamidin (and higher carthamin) at this harvest. Row spacing influenced the content of carthamidin, and higher contents were measured in petals at high plant density (50 plants m^−2^) at the second harvest. Low plant spacing (thus high plant population) would counteract the deteriorative effect of high light intensity upon colorants (Pu et al., 2019). Steberl et al. (2020) recommended a plant density of 40 plants m^−2^ to achieve maximum floret and carthamidin yields under the conditions in Southwest Germany.

Carthamin extracted from safflower petals in this study was on average 0.34% and 0.45% at the first and second harvests, respectively, which is in line with the content reported for this species by Ghorbani et al. (2015) but much higher than the content reported by Emongor and Setshogela (2024). Carthamin contents ranging from 0.02% to 6% have been reported, depending on genotype and the time of petal harvest (Emongor and Setshogela, 2024). Significant “plant density” effect at the first harvest and both significant “sowing time” and “plant density” effects at the second harvest suggest the possibility of manipulating the content of carthamin in petals through agronomic management. Carthamin is synthesized from carthamidin via oxidation, which may explain the opposite trend of the two pigments described in the present study at the second harvest. According to Emongor and Setshogela (2024), who reported a lower carthamin content in petals at the initial stages of flowering, in the present study, carthamin was lower (−24%) at the first harvest, irrespective of sowing time.

The free radical scavenging activity of safflower petal extracts was assessed using DPPH. Lower antioxidant activity was measured at the second harvest as a probable effect of lower phenol and flavonoid contents, as also confirmed by the strict correlation found among these traits. Phenolic compounds have been extensively reported as the major contributors to the antioxidant activity of fruits and vegetables (Jacobo-Velázquez and Cisneros-Zevallos, 2009; Patanè et al., 2025). A similar decreasing trend of radical scavenging activity was reported by Salem et al. (2011) in safflower petals during flowering. Different than the findings of Al-Nafei and Al-Mohammad (2021), who reported a lowering of the antioxidant activity in safflower petals as planting distance was increased from 10 (high plant density) to 20 cm (low plant density), in this study, the free radical scavenging activity of petal extracts was reduced at higher plant density at the first harvest and did not change with plant population at the second harvest. High antioxidant activity has been reported in literature for carthamin, similar to that of vitamin C and much higher than that of carthamidin (Hiramatsu et al., 2009; Salem et al., 2014); however, in this study, the antioxidant activity was positively correlated only with carthamidin.

Interesting correlations were found among the examined traits. The positive correlation of crude protein *vs*. total phenols, flavonoids, carthamidin, and antioxidant activity revealed how the nutritional value of petals is positively associated with that nutraceutical. The positive correlations of the DPPH scavenging activity *vs*. total phenols, flavonoids, and carthamidin also indicate that in safflower, the antioxidant capacity of the petals is maximized in the early stages of flowering when the yellow pigment achieves its highest level. However, different than what was reported by Hiramatsu et al. (2009), a negative correlation of red pigment carthamin *vs*. the antioxidant activity was found, probably because the levels of carthamidin at both harvests were high, but those of carthamin were low, with petals harvested at flowering when petals were still moist. Indeed, carthamin is maximized at the senescence of petals (dry petals) (Hiramatsu et al., 2009; Pu et al., 2021).

In PCA, the positioning of all sowings and plant densities with traits measured at the first harvest in the right part of the graph, having positive values of PC1, and all sowings and plant densities with traits measured at the second harvest in the left part of the graph, having negative values of PC1, indicates that petal production and quality may greatly vary with the time of harvest.

## 5 Conclusions

The results of this study revealed that sowing in December may provide higher head and petal yields than later winter sowings in January and February of safflower grown in the semi-arid regions of Southern Italy. In this regard, safflower can be a valid alternative to introduce into the typically cereal-based cropping systems of these regions. Moreover, overall, greater contents in phenols and flavonoids, and pigment carthamidin, which all have a great scavenging activity against free radicals, and minor changes in the proximate composition of petals can be achieved with the adoption of sowings in late fall–early winter. Increased plant density from 25 to 50 plants m^−2^ may be profitable when later winter sowings in January or February are imposed since a high plant population may compensate for the yield loss. Indeed, both heads and petals per unit area were increased with the increase in plant density, with no or minor effects upon nutrients, nutraceuticals, and pigments.

Harvesting at late flowering (~90% flowers open) resulted in greater yields but led to an overall lower quality (in terms of both nutrients and nutraceuticals) of petals; this was maximized at the onset of flowering (~30% flowers open). However, considering the potential application of safflower pigments as natural colorants in the agri-food industry, a 27% increase in carthamidin yield and a 92% increase in carthamin yield per hectare obtained at the second harvest (**Table 7**) indicate that shifting the petal harvest to late flowering may be more profitable. Therefore, early harvest for quality and late harvest for petal and pigment yields are recommended, suggesting that the choice of harvest time of petals strictly depends on the specific trait desired.

**Table 7:** Yields in carthamidin and carthamin in safflower cv. Catima at the two petal harvests.

Harvest	Petals	Carthamidin		Carthamin
	kg ha^−1^ DW				
1^st^	310.4	26.06	1.05	
2^nd^	454.0 (+46%)	32.98 (+27%)	2.02 (+92%)	

DW, dry weight.

These results evidenced the potential of safflower petals as a suitable source of natural bioactive compounds and pigments. Taking into consideration their great antioxidant activity, safflower petals can also be used as a healthy food ingredient. However, despite several potential industrial applications of its petals, safflower is still underutilized. Further studies are recommended to evaluate how other agronomic practices (e.g., fertilization) could influence the petal yield and quality of this neglected crop.

It is important to emphasize that these conclusions are based on a single safflower cultivar, and the responses to sowing time and plant density may vary across different genotypes. Therefore, further investigations involving multi-year and multi-genotype studies are recommended to strengthen the current findings.

## Data Availability

The raw data supporting the conclusions of this article will be made available by the authors, without undue reservation.
